# Sequencing confirms *Anopheles stephensi* distribution across southern Yemen

**DOI:** 10.1186/s13071-024-06601-1

**Published:** 2024-12-18

**Authors:** Yasser A. Baheshm, Alia Zayed, Abdullah A. Awash, Madison Follis, Payton Terreri, Jeanne N. Samake, Adel Aljasari, James F. Harwood, Audrey Lenhart, Sarah Zohdy, Samira M. Al-Eryani, Tamar E. Carter

**Affiliations:** 1 National Malaria Control Program, Ministry of Health, Aden, Yemen; 2 US Naval Medical Research Unit-EURAFCENT (Previously NAMRU-3), Cairo Detachment, Cairo , Egypt; 3 World Health Organization, Country Office, Sana’a, Yemen; 4 Department of Biology, Baylor University, Waco, TX USA; 5 Entomology Branch, U.S. Centers for Disease Control and Prevention, Atlanta, GA USA; 6 Medical Research Unit-EURAFCENT (Previously NAMRU-3), US Naval, Sigonella, Italy; 7 Department of Universal Health Coverage/Communicable Diseases Prevention and Control, Eastern Mediterranean Regional Office, World Health Organization, Cairo, Egypt

## Abstract

**Abstract:**

The invasion of *Anopheles stephensi* in Africa warrants investigation of neighboring countries. In this study, genetic analysis was applied to determine the status of *An. stephensi* in southern Yemen. Cytochrome *c* oxidase subunit I (*COI*) and internal transcribed spacer 2 (ITS2) were sequenced in *An. stephensi* collected in Dar Sa’ad (Aden City), Tuban, Rodoom, Al Mukalla, and Sayhut, and phylogenetic analysis confirmed *An. stephensi* identity. Our analyses revealed that the ITS2 sequences were identical in all *An. stephensi*, while *COI* analysis revealed two haplotypes, one previously reported in northern Horn of Africa and one identified in this study for the first time. Overall, these findings revealed low levels of mitochondrial DNA diversity, which is consistent with a more recent population introduction in parts of southern Yemen relative to the Horn of Africa. Further, whole genomic analysis is needed to elucidate the original connection with invasive populations of *An. stephensi* in the Horn of Africa.

**Graphical Abstract:**

**Figure:**
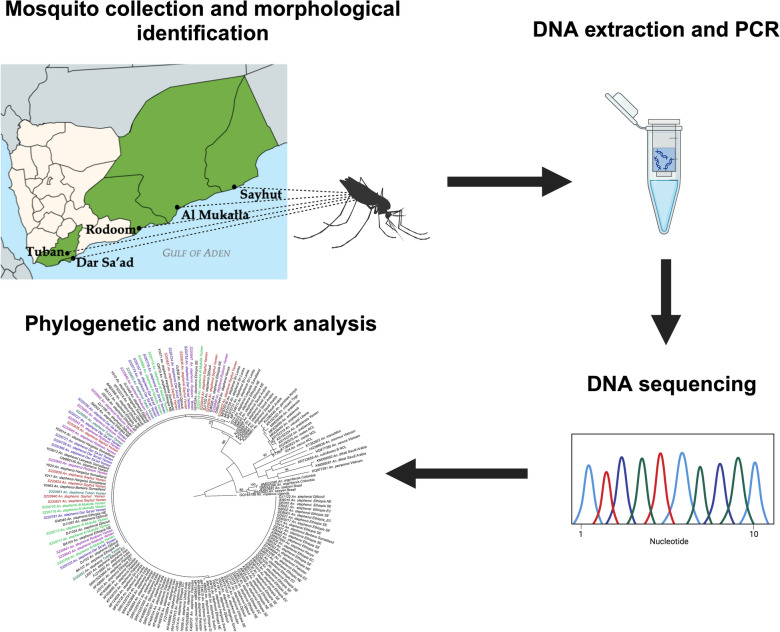

**Supplementary Information:**

The online version contains supplementary material available at 10.1186/s13071-024-06601-1.

## Background  

*Anopheles stephensi* is a malaria vector originally found in South Asia and the Middle East. In 2012, *An. stephensi* was detected on the African continent in Djibouti City [1] and 3 years later in Kebridehar, Ethiopia [2]. With the detection of the vector in East Africa and parts of West Africa [3], there is rising concern that this invasive vector could spread further. The connection between the presence of *An. stephensi* and malaria outbreaks in Djibouti City, Djibouti [4] and Dire Dawa, Ethiopia [5] heightens the need to track the spread of this invasive species.

Given the proximity to the Horn of Africa (HoA), the status of *An. stephensi* in the Arabian Peninsula is of growing interest. The predicted native range of *An. stephensi* includes Saudi Arabia and Oman [6]. However, *An. stephensi* has not been reported in Yemen until recently. *An. stephensi* was detected in Yemen in 2021 in Aden City, located in the southern part of Yemen [7]. Aden City shares some similarities with locations where *An. stephensi* is now well established, which serve as major hubs for its onward spread across the HoA [8]. It is an urban area and port city with frequent population movement inward through the flow of internally displaced persons and refugees from the Horn of Africa [9]. It is a major port city connected to Bosaso, Somalia and Mukalla Port City, another location on the southern coast of Yemen with a recent detection of *An. stephensi*. Previous analysis of cytochrome oxidase 1 (COI) has indicated high connectivity and likely recent ancestry between northeastern Ethiopia to Lawyacado, just 20 km away from a major port in Djibouti City, demonstrating population dissemination from a port city inland (Samake et al., 2022). Finally, an increase in malaria incidence has also been reported in Aden, similar to outbreaks observed in major hubs of *An. stephensi* populations such as Dire Dawa, Ethiopia and Djibouti City, Djibouti [9]. Given the detection of *An. stephensi* in these two locations in the southern part of Yemen, it is important to confirm how far *An. stephensi* has spread throughout the south.

Molecular confirmation of species identification is an important component of the tracking of invasive *An. stephensi* across its invasive range [10]. It provides a basis to evaluate the successful integration of the updated morphological keys that include *An. stephensi* into ongoing vector surveillance. In addition, DNA sequence-based molecular identification has the benefit of providing preliminary insight into the relationship between newly identified invasive populations and other reported populations. This study reports the results of molecular analyses of morphologically identified *An. stephensi* across southern Yemen, including four newly reported locations.

## Methods

### Site descriptions

**Fig. 1:**
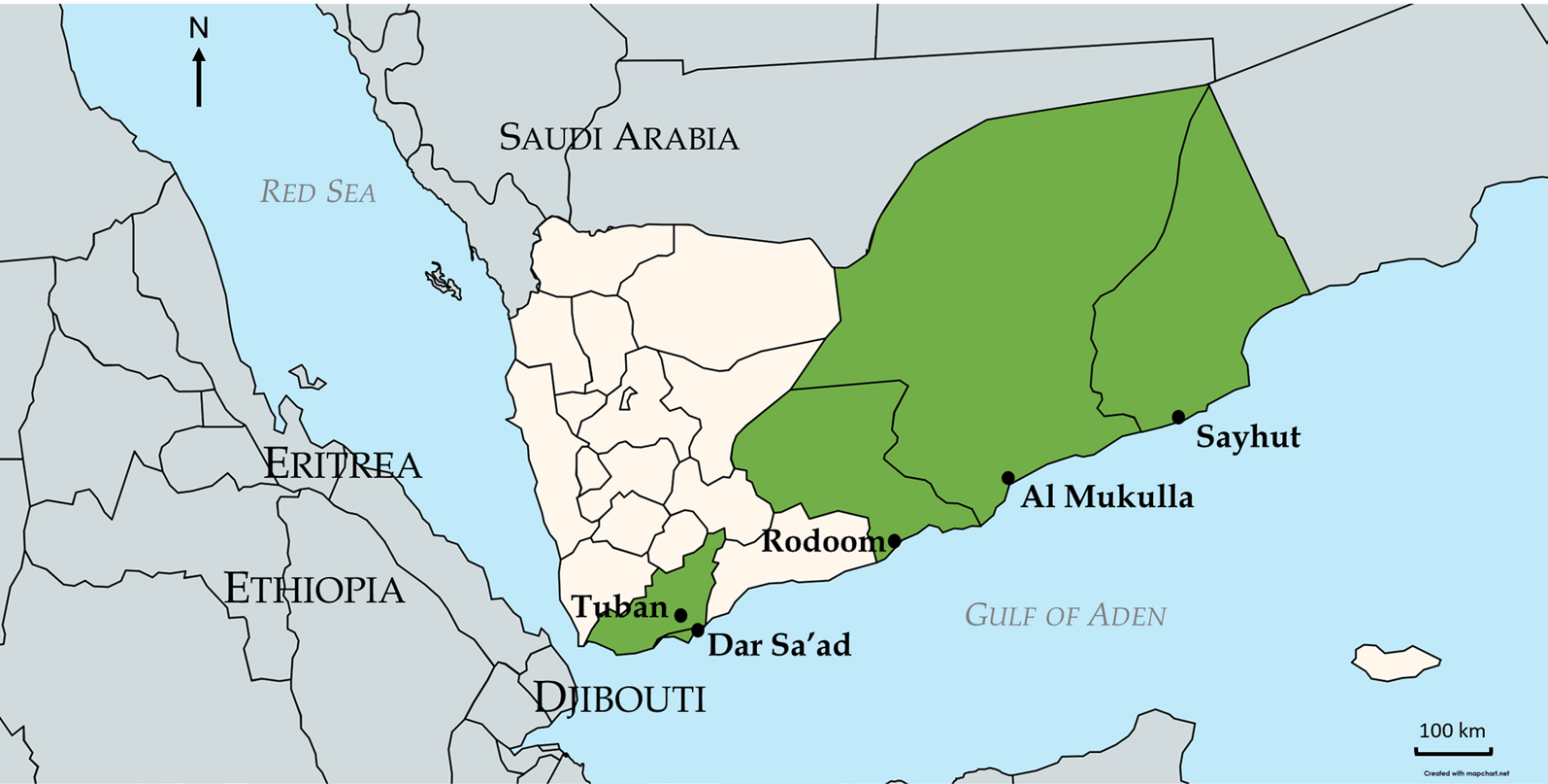
Map of collection sites created using MapChart and edited with Microsoft PowerPoint

A total of five sites were surveyed for this study (Table 1; Fig. 1): Dar Sa’ad (Aden City), Tuban in Lahj governorate, Rodoom in Shabwah governorate, Al Mukalla Port City in Hadramout governorate, and Sayhut in Al Mahrah governorate. Aden is a major port city along a historic maritime route connecting the Horn of Africa and the Arabian Peninsula. The climate in Aden is mostly desert, although there is steady humidity year round. Tuban is located just under 40 km north of Aden with similar climate. Rodoom coastal city in Shabwah governorate is located between Hadramout to the east and Abyan to the west with coastal and semiarid climate. Al Mukalla port city is located over 500 km east of Aden and has a coastal and semi-arid climate. Sayhut is the easternmost site of this collection and has a subtropical climate with peak rains in May.

**Table 1:** Description of sites

Site	Governorate	Coordinates	Date collected	Climate
Dar Sa’ad	Aden	12.78652104214733, 45.01848534347771	October, December 2021	Desert
Tuban	Lahj	13.05807428525727, 44.88331315931494	December 2021	Desert
Rodoom	Shabwah	13.841611, 47.59567	June 2022	Coastal and semi-arid
Al Mukalla	Hadramout	14.54943802880376, 49.12458936878869	October 2021, April, September, October 2022	Coastal and semi-arid
Sayhut	Al Mahrah	15.202806, 51.201750	May 2022	Subtropical

### Collections

Larvae were collected using the dipping method over the course of 3 days and reared to adult stage. Potential breeding habitats surveyed included domestic containers, air conditioning drips, accumulated water in brick factories, and cemeteries (Supplemental Fig. 1). Adult *Anopheles* were identified morphologically using the Afrotropical mosquito key [11]. The specimens morphologically identified as *An. stephensi* were preserved with silica gel and a subsample of specimens was sent to Baylor University for molecular analysis.

### PCR and sequence analysis

Two loci were selected for polymerase chain reaction (PCR) amplification-based species identifications: cytochrome *c* oxidase subunit 1 (*COI)* and internal transcribed spacer 2 (ITS2) as previously applied [2, 12]. First, species were confirmed using a PCR species-specific assay targeting the ITS2 region [2, 13] and with PCR and sequencing of the ITS2 and an informative portion of *COI* using methods described previously [2]. The primer sequences for PCR in the ITS2 endpoint assay are 5.8SB (5′-ATCACTCGGCTCGTGGATCG-3ʹ) and 28SC (5ʹ- GTCTCGCGACTGCAACTG-3ʹ) [13]. The primer sequences for the universal ITS2 PCR for sequencing were 5.8SB (5ʹ-ATCACTCGGCTCGTGGATCG-3ʹ) and 28SB (5ʹ-ATGCTTAAATTTAGGGGGTAGTC-3ʹ) [13]. The primer sequences for *COI* PCR are LCO1490F (5ʹ-GGTCAACAAATCATAAAGATATTGG-3ʹ) and HCO2198R (5ʹ-TAAACTTCAGGGTGACCAAAAAATCA-3ʹ) [14]. ITS2 and *COI* PCR products were sequenced using Sanger sequencing technology. Sequences were then trimmed and analyzed using CodonCode Aligner (CodonCode Corporation, Centerville, MA). Final sequences were submitted to the National Center for Biotechnology Information’s (NCBI) Basic Local Alignment Search Tool (BLAST) (accession nos.: PP752283, PP752284, PQ006012). Alignments were generated in CodonCode Aligner that included previously published sequences from NCBI GenBank. ITS2-based sequence identification excluded the microsatellite region found within ITS2 [10]. Likewise, only a portion of the *COI* previously identified as geographically informative was used for subsequent analysis to maximize the overlap with existing datasets for haplotype and phylogenetic comparisons (see Carter et al. 2021). *An. stephensi* sequences from NCBI GenBank were included in the *COI* phylogenetic analysis: 12 from Djibouti, 8 from India, 2 from Sudan, 46 from Ethiopia, 2 from Kenya, 13 from Pakistan, 7 from Saudi Arabia, 8 from Sri Lanka, 1 from the United Arab Emirates, 11 from Somaliland, and 2 from Iran. Sequences were also included from some other *Anopheles* species to support species identification with an outgroup of *An. implexus* (GQ165788). Phylogenetic analysis was conducted using the maximum-likelihood (ML) method with RAxML [15]. The best scoring trees under ML with bootstrap values from RAxML were viewed and annotated using Figtree [16]. We also created a minimum spanning network based on COI sequences to evaluate the relationship and distribution of haplotypes among the Yemen *An. stephensi* and those along the native and invasive range.

## Results and discussion

A total of 44 mosquitoes were molecularly characterized and all specimens were confirmed to be *An. stephensi* with the ITS2 end-point assay. In addition, BLAST analyses of the *COI* and ITS2 sequences revealed the highest scoring matches for *An. stephensi*. Most of the *COI* sequences had a 100% sequence identity match with *An. stephensi* sequences originating from northeastern Ethiopia, Somaliland, Djibouti, and Yemen. The 162 bp segment of the ITS2 sequence were identical for all specimens and had a 100% sequence identity match with other *An. stephensi* sequences. Phylogenetic analysis confirmed *An. stephensi* identity (ITS2 bs = 100) (Supplementary Fig. 2).

**Fig. 2:**
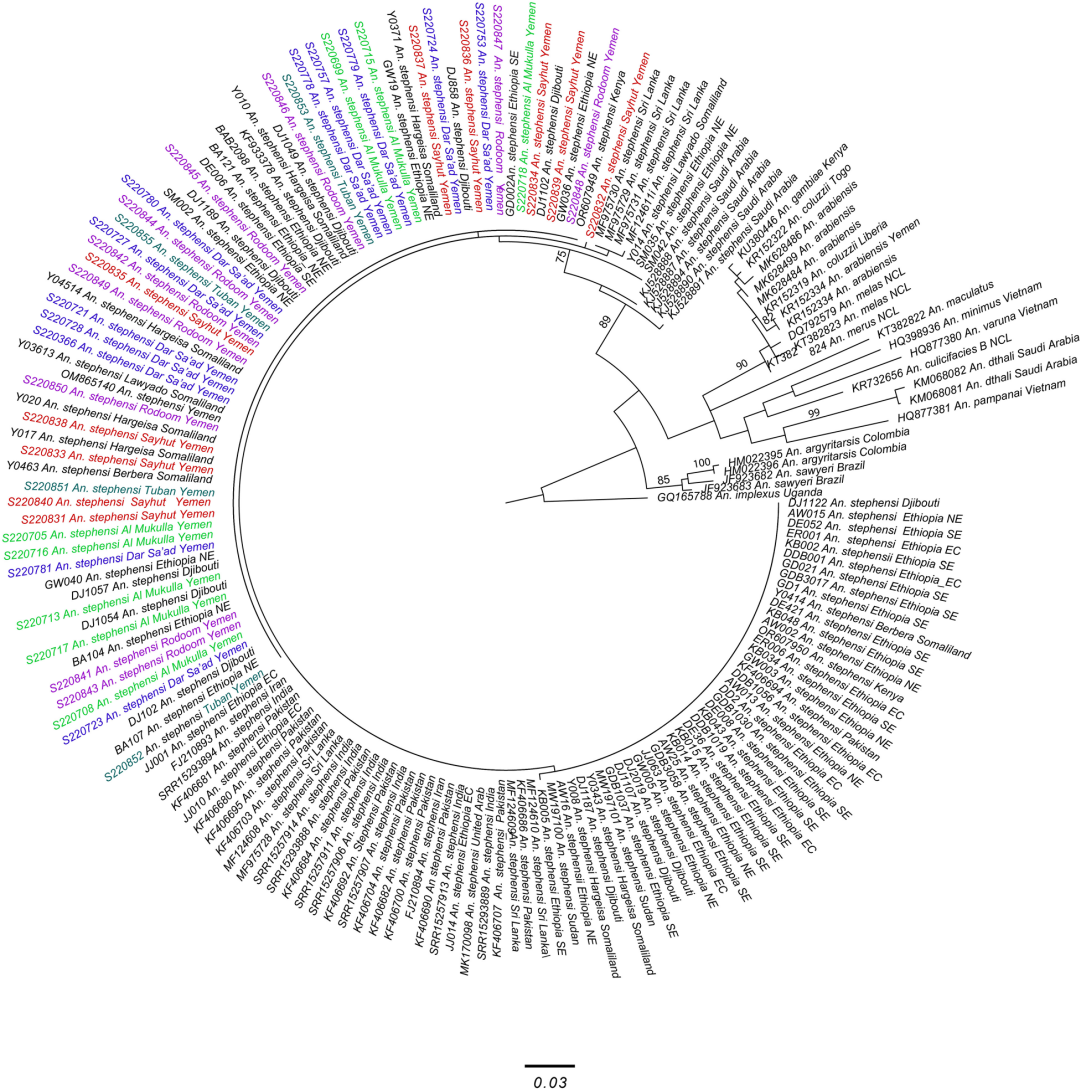
Phylogenetic analysis of *An. stephensi COI* sequences from southern Yemen using the maximum likelihood approach. Yemen sequences are in color. Rodoom, purple; Sayhut, red; Mukalla, light green; Dar Sa’ad (Aden), blue; Tuban, teal

Analysis of a specific region of the *COI* sequence has previously been identified as geographically informative [17]. Using this region of the *COI* gene, we identified two haplotypes among the Yemen samples. One haplotype was found in the majority of the mosquitoes (*n* = 43) (Supplementary Fig. 3). This haplotype has been observed in Yemen and in the northeastern Horn of Africa [7, 17, 18] in other studies and designated Hap3 by Carter et al. [17]. The second haplotype (*n* = 1, found in Sayhut) has not been reported previously. As the second unique Yemen haplotype, it will be designated HapYem2 in this study. The *COI* phylogenetic tree provided additional insight into the relationship between southern Yemen and other areas in the *An. stephensi* range (Fig. 2). Phylogenetic analysis showed significant bootstrap support for *An. stephensi* differentiation from Saudi Arabia for all sequences, although further geographic distinctions were not detected in this phylogenetic tree, likely due to the low variation of this marker.

While there was no significant bootstrap support, *An. stephensi* populations in southern Yemen seem to be somewhat distinguished from *An. stephensi* in the native range (Pakistan, India, Saudi Arabia, United Arab Emirates, and Iran), as well as some portions of the invasive range in the Horn of Africa (Sudan, eastern central Ethiopia, southeastern Ethiopia,). This may suggest a stronger connection to older *An. stephensi* populations in the Horn of Africa such as those from Djibouti, Somaliland, and northeastern Ethiopia. While there is connection to these older populations, overall, the populations observed in this study are more homogeneous relative to populations across eastern Ethiopia, Djibouti, and Somaliland. This provides support for a more recently established population of *An. stephensi* in southern Yemen compared with those in northern Horn of Africa populations. Interestingly, the *An. stephensi* population in the eastern most site, Sayhut, contained a singular haplotype that is not present elsewhere. This could be suggestive of another entry or longer established population, however, a larger sample size and investigation into other regions in the genome would be needed to make a conclusion.

Overall, phylogenetic analysis of the *COI* gene revealed that the relatively homogeneous population of southern Yemen is likely a recent establishment that is highly connected to longer-established invasive populations in northeastern Ethiopia, Djibouti, and Somaliland. There are limitations to these conclusions without full representative sampling of *An. stephensi* across the native  geographic range in the Arabian Peninsula and by the limited resolution of the markers used in this study. Additional genome-wide analyses are needed to establish the origin of the *An. stephensi* in Yemen. Regardless, these findings highlight the need for continued molecular surveillance in this region.

## Supplementary Information

**Supplementary materials 1.**

## Data Availability

Sequence data that support the findings of this study have been deposited in the National Center For Biotechnology Information Genbank (Accession no. PP752283, PP752284, PQ006012).
