# Supplementary material for: Sequencing confirms Anopheles stephensi distribution across southern Yemen
Source: Parasit Vectors. 2024 Dec 18;17:507. doi: 10.1186/s13071-024-06601-1 (PMC11657292; doi:10.1186/s13071-024-06601-1)
Supplement: Supplementary file 1 — Supplementary materials 1. [file 13071_2024_6601_MOESM1_ESM.pdf]

Supplemental Information

Figures

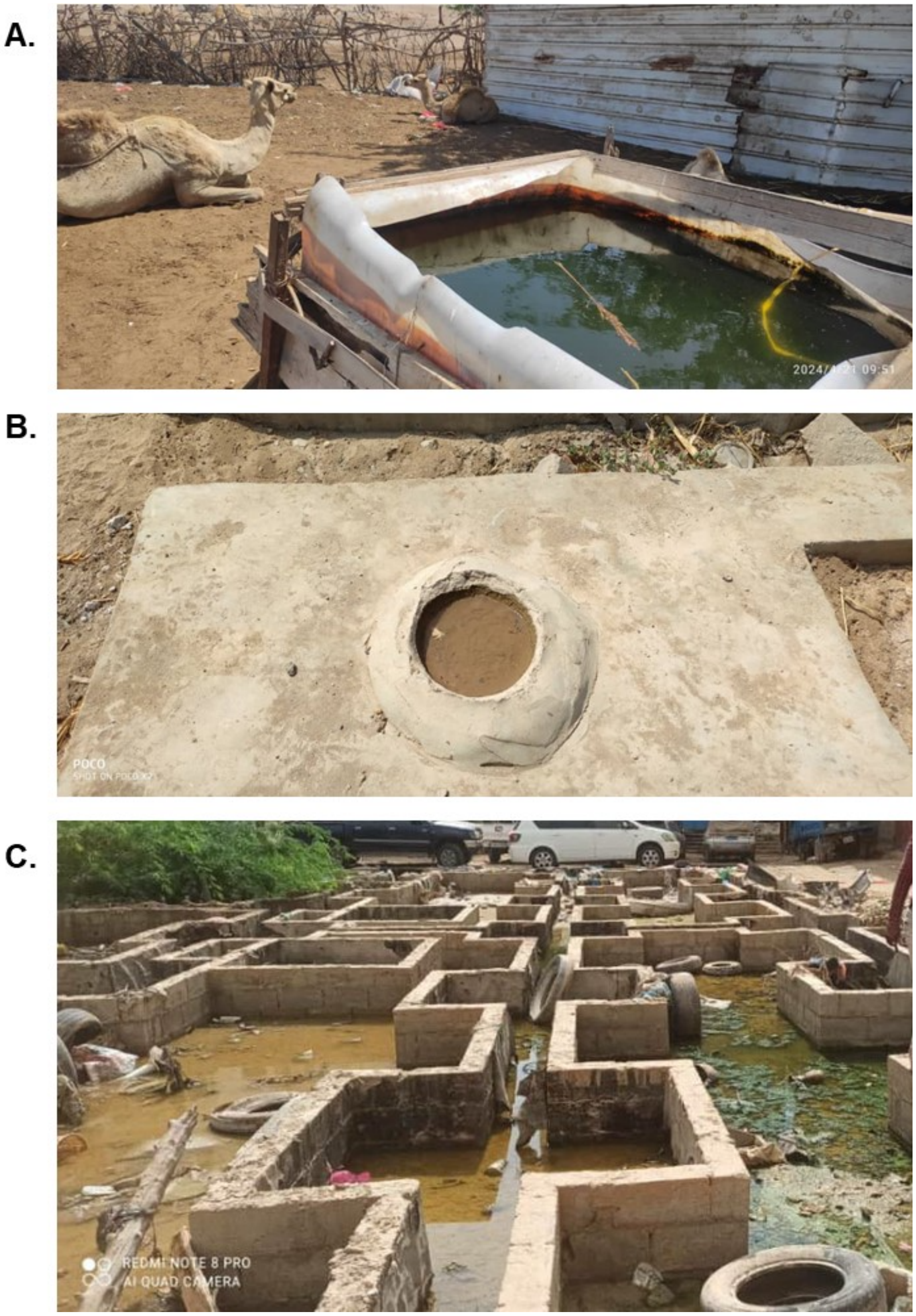

Supplemental Figure 1. Example of breeding habitats where *An. stephensi* were collected, including (A) water reservoir, (B) flower beds on grave filled with water, and (C) brick factory. Photos captured by National Malaria Control Programme in Aden, Yemen.

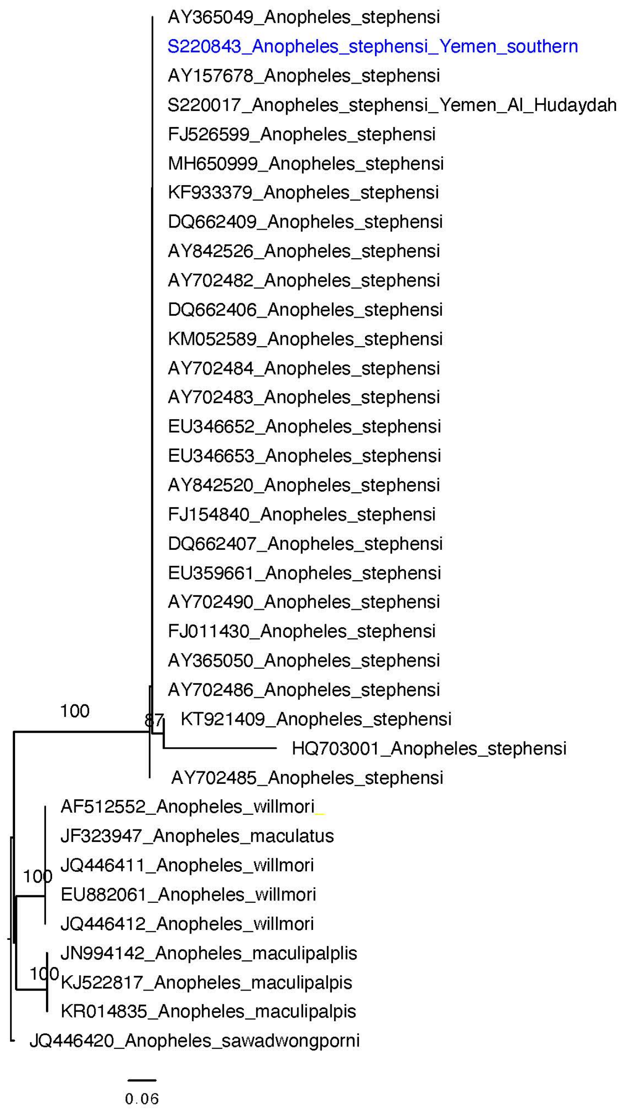

Supplemental Figure 2. Phylogenetic analysis of *An. stephensi* ITS2 sequences from southern Yemen using the maximum likelihood approach. The ITS2 sequence observed in southern Yemen is in blue.

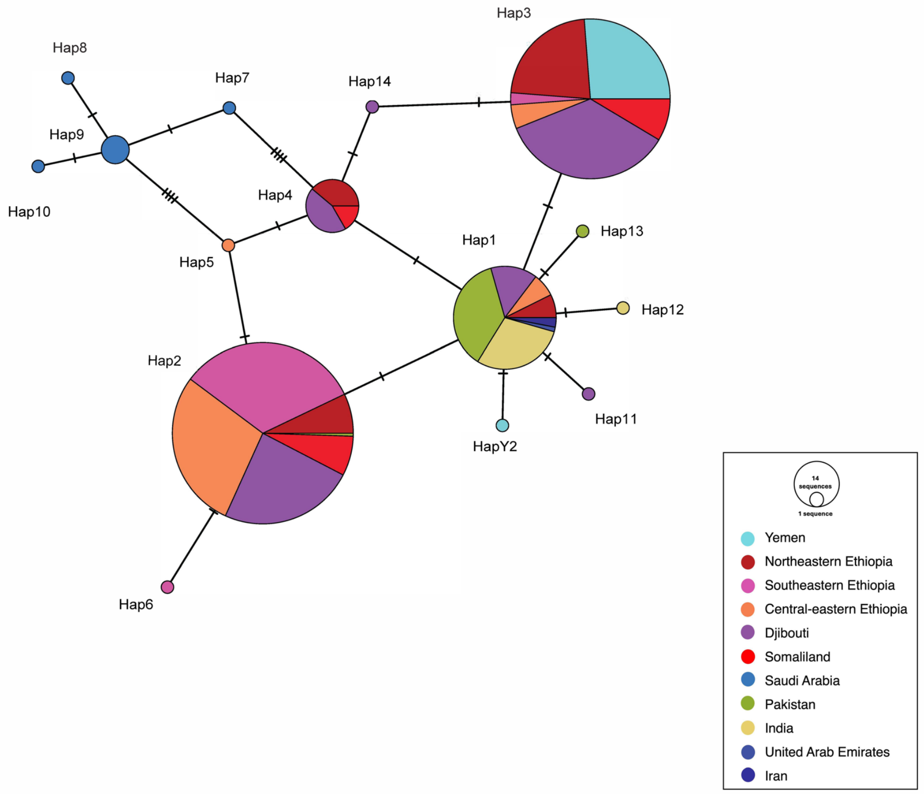

Supplemental Figure 3. Minimum spanning network of *An. stephensi* cytochrome *c* oxidase subunit 1 sequences from Yemen with other sequences from across the *An. stephensi* geographical range.
